# Hybrid Porous Nanomaterials for Energy and Environment

**DOI:** 10.3390/nano12142471

**Published:** 2022-07-19

**Authors:** Evangelos P. Favvas

**Affiliations:** Institute of Nanoscience and Nanotechnology, NCSR “Demokritos”, 15341 Agia Paraskevi, Greece; e.favvas@inn.demokritos.gr

Porous materials have applications in a wide range of research and industrial processes. The food industry, agricultural products, biomedical and pharmaceutical technology, wood processing, urban construction, ceramic products, gas separation, filtration processes, drying, and catalysts are just a few examples. The scientific interest has increased in the investigation of porous nanomaterial properties—especially the properties of hybrid porous nanomaterials.

Hybrid nanomaterials contain two or more different components, typically, inorganic and organic components, which are brought together by specific interactions, and the result is the synergistic enhancement of their chemical and functional properties. These new enhanced properties have piqued the interest of both academia and the industry towards energy, environmental, and health applications. Hybrid nanoporous materials can provide solutions in numerous applications, such as fuel cells, batteries, sensors and biosensors, building materials, gas separation and storage processes, catalytic reactions, and water treatment processes, to name a few.

Within this context, this Special Issue, entitled “Hybrid Porous Nanomaterials for Energy and Environment”, offers to readers a compilation of relevant contributions exhibiting the potentialities of emerging hybrid porous nanomaterials with potential applications bilaterally in energy and the environment.

The current Special Issue consists of six original research papers in different, but equally interesting, fields of porous nanomaterials with remarkable applications in energy and environmental fields. A short description for each article of the Special Issue “*Hybrid Porous Nanomaterials for Energy and Environment*” is presented herein.

A study which describes the best coating configuration for nanoparticles of a porous metal–organic framework (MOF) onto both insulating or conductive threads and nylon fiber is part of this collection. In this study, by Rauf et al., customized polymethylmethacrylate sheets (PMMA) holders to deposit MOF layers onto the threads or fiber using the LB technique were designed and fabricated. The results demonstrate a significant contribution in terms of MOF monolayer deposition onto single fiber and threads that will contribute to the fabrication of single fiber or thread-based devices in the future [1].

Heng et al. studied the solar-powered photodegradation of pollutant dyes using silver-embedded porous TiO_2_ nanofibers. Remarkable evidence of this work is that the use of electrospun Ag-embedded TiO_2_ nanofibers, as a photocatalyst, was effective in decomposing rhodamine B and methyl orange dyes under a solar simulator in 3 h. This showcases the potential of a simple and economic wastewater treatment system for the removal of organic pollutants using hybrid porous nanomaterials [2].

An effective self-supported electrode for the electrocatalytic hydrogen-evolution reaction was prepared and presented by Qi et al. This electrode was a novel porous three-dimensional Ni-doped CoP_3_ nanowall array on carbon cloth. The synthesized/studied samples exhibited rough, curly, and porous structures, which are beneficial for gaseous transfer and diffusion during electrocatalytic processes. The achieved over-potentials of 176 mV for the hydrogen-evolution reaction afforded a current density of 100 mA/cm^2^, indicating that the electrocatalytic performance could be dramatically enhanced via Ni doping. The Ni-doped CoP_3_ electrocatalysts with increasing catalytic activity should have significant potential in the field of water splitting into H_2_. As the authors mentioned, their study opens avenues for the further enhancement of electrocatalytic performance through tuning of the electronic structure and d-band center by doping [3].

Another interesting study was performed by Alinejad et al., and concerns two porous silica membranes, a novel structure of a hydrogen–membrane reactor coupling HI decomposition and CO_2_ methanation. A 2D model was developed, and the effects of feed flow rate, sweep gas flow rate, and reaction pressure were examined by computational fluid dynamic (CFD) simulation. The theoretical predictions demonstrated that the best results in terms of HI conversion were 74.5% for the methanation membrane reactor and 67% for the simple membrane reactor [4].

In another study, conducted by Metaxa et al., the effect of different MWCNT concentrations and different types of surfactants and a superplasticizer were examined to reinforce, at the nanoscale, a white cement mortar typically used for the restoration of monuments of cultural heritage. Carboxylation of the MWCNT surface with nitric acid did not improve the mechanical performance of the white cement nanocomposites. The parametric experimental study showed that the optimum combination of 0.8 wt.% of cement superplasticizer and 0.2 wt.% of cement MWCNTs resulted in a 60% decrease in the electrical resistivity; additionally, the flexural and compressive strengths were both increased, by approximately 25% and 10%, respectively [5].

Finally, the sixth paper, by Tolkou et al., examined the performance of activated carbon produced from coconut shells, modified by lanthanum chloride, for Cr(VI) removal from waters. The structure of the formed material (COC-AC-La) was characterized by the application of BET, FTIR, and SEM techniques. The results indicated that the maximum Cr(VI) removal was observed at pH 5; a 4 h contact time and 0.2 g/L dosage of adsorbent was adequate to reduce Cr(VI) from 100 μg/L to below 25 μg/L. The maximum adsorption capacity achieved was 6.3 μg/g at pH 5. At this pH value, the removal percentage of Cr(VI) reached 95% for an initial Cr(VI) concentration of 30 μg/L. At pH 7, the corresponding efficiency was roughly 60%, resulting in residual Cr(VI) concentrations below the anticipated drinking water limit of 25 μg/L of total chromium, when the initial Cr(VI) concentration was 50 μg/L. Consecutive adsorption and regeneration studies were also conducted, and the results showed a 20% decrease in adsorption capacity after five regeneration cycles of operation [6].

At this point, I would like to thank the authors for choosing the Special Issue “Hybrid Porous Nanomaterials for Energy and Environment” for submitting their excellent studies. In fact, the collection of these articles took place during the COVID-19 pandemic, a strange period where the laboratories, worldwide, were working in non-normal conditions.

I also hope, and wish, that all readers will enjoy the articles of this Special Issue.
